# The Preparation of Matches Free from Phosphorus

**Published:** 1865-01

**Authors:** 


The Preparation of Matches free from Phosphorus.—Hierpe has published (*Polytech. Centralblatt*, 1864, p. 696) the following receipts for a composition for the heads of matches, and for an igniting surface. That for the matches is as under:—

| | |
|---|---|
| Chlorate of potash . . . | 4 to 6 parts |
| Bichromate of potash . . . | 2 " |
| Ferric oxide . . . . . . | 2 " |
| Strong glue . . . . . . | 3 " |

Oxide of iron may be replaced by oxide of lead or of manganese. The above preparation will not ignite on sand-paper, but requires a surface specially prepared for it, and the author employs the following on the boxes:—

| | |
|---|---|
| Sulphide of antimony . . | 20 parts. |
| Bichromate of potash . . | 2 to 4 " |
| Oxide of iron, lead, or manganese . | 4 to 6 " |
| Glass powder . . . . . | 2 " |
| Strong glue or gum . . . | 2 to 3 " |

Another composition is described by Dr. H. Poltzer (*Polytech. Centralblatt*, 1863, p. 1642.) A solution of sulphate of copper is divided into two equal parts—one is supersaturated with ammonia, the other with hyposulphite of soda. The two solutions are now mixed, and the mixture briskly stirred. A violet-colored powder now deposits, which is a compound, says the author, of hyposulphurous acid with oxide and suboxide of copper, soda and ammonia. A mixture of this salt with chlorate of potash detonates when struck with a hammer, and when rubbed in mortar ignites and burns like gunpowder, leaving a black residue.

The above salt the author proposes to use for matches. It is not soluble in water, and the mixture with chlorate of potash is not hygroscopic. The mixture may be made with moist chlorate and the gum solution, and can be safely dried at 50° C. or higher. It inflames when rubbed on a rough surface, and the temperature developed is sufficiently high to ignite sulphur on the stick.

The only difficulty the author finds is in making the mass coherent: when dried on the stick he found that it would crack and drop off when rubbed. A manufacturer will probably soon overcome this difficulty.

The proportions made use of were one part of the copper salt, and two parts of chlorate mixed in a sieve, and then made into a mass with solution of gum, together with a little glass powder. This mixture was applied to matches dipped in sulphur as usual.—*Am. Jour. of Pharmacy, from London Chem. News, Dec.* 10, 1864.

